# Association of Proton Pump Inhibitors on Psoriasis Treatment and Development: A Systematic Review

**DOI:** 10.1177/12034754241265711

**Published:** 2024-07-26

**Authors:** Siddhartha Sood, Ryan Geng, Samantha Bestavros, Khalad Maliyar, Muskaan Sachdeva, Asfandyar Mufti, Jensen Yeung

**Affiliations:** 1 Temerty Faculty of Medicine, University of Toronto, Toronto, ON, Canada; 2 Division of Dermatology, Department of Medicine, University of Toronto, Toronto, ON, Canada; 3 Department of Dermatology, Sunnybrook Health Sciences Centre, Toronto, ON, Canada; 4 Probity Medical Research, Waterloo, ON, Canada; 5 Department of Dermatology, Women’s College Hospital, Toronto, ON, Canada

**Keywords:** psoriasis, proton pump inhibitor, cutaneous adverse event, treatment, systematic review

To the Editor,

Proton pump inhibitors (PPIs) are well-established for treatment of gastroesophageal reflux disease and peptic ulcer.^1^ While novel, use of these therapies for psoriasis patients has been reported with additional studies evaluating development of psoriasis on PPI therapy.^1-3^ This systematic review aims to examine evidence regarding these associations.

We followed Preferred Reporting Items for Systematic Reviews and Meta-Analyses (PRISMA) guidelines to search Embase and MEDLINE databases using specific keywords (Supplemental Table 1). Quality of evidence was assessed using Oxford Centre for Evidence-Based Medicine 2011 Levels of Evidence. After independent screening by 2 reviewers, 10 articles (publication date: 2000-2021) reflecting 3092 patients were included (Supplemental Figure 1; Supplemental Table 2). The mean age was 50.4 years (range: 28-59 years) with sex reported in 2991 (96.7%) patients (male: 59.7%, 1787/2991; female: 40.2%, 1204/2991). Types of psoriasis documented were plaque (97.7%, 376/385), guttate (1%, 4/385), pustular (0.8%, 3/385), and palmoplantar (0.5%, 2/385). *Helicobacter pylori* infection was confirmed in 478 (15.5%) patients.

Outcomes were classified into the following categories: *de novo* psoriasis (93.5%, 2890/3092), improvement of psoriasis (6%, 192/3092), and no effect of PPI treatment on psoriasis (2.6%, 10/3092). The specific PPIs utilized were reported in 2533 (81.9%) instances, most commonly being lansoprazole (35.5%, 900/2533), omeprazole (29.4%, 745/2533), and esomeprazole (26.5%, 670/2533; Supplemental Table 3). Across 2 (20%) studies that reported on *de novo* psoriasis, odds ratios (ORs) were between 1.52 and 1.54 (highest incidence with lansoprazole; OR 1.25) and the highest adjusted hazard ratio was 2.6 (*P* < .01; Supplemental Table 2).^1,2^ Among 8 (80%) studies reflecting 202 patients with reported outcomes of PPI use for psoriasis, complete clearance and partial resolution were achieved in 2 (1%) and 190 (94%) of cases, respectively (mean treatment duration: 15 days; 202/3092). Of these, a lack of resolution was observed in 10 (5%) patients with omeprazole use. The mean percent improvement from baseline in Psoriasis Area and Severity Index (PASI) was 58.9% [reported in 171/3092 (5.5%) of cases], with 63.6% (6/11) of reported patients achieving PASI 90 (Supplemental Table 3). Concurrent systemic medications and/or phototherapy for psoriasis were used in 153 (79.7%) of PPI-treated cases, majority being apremilast (26%, 50/192; Supplemental Table 2).

While the relationship between psoriasis and PPI use remains unclear, neutrophilic infiltrates along with glandular mucosal changes have been identified in patients with psoriasis.^4^ Furthermore, it has been postulated that certain gut microbiota may induce the Th17 immune pathway.^5^ As PPIs can mediate gastrointestinal inflammation and alter the microbiome balance, this may be a mechanism by which psoriasis may occur or improve with therapy. A population-based study in Taiwan identified an adjusted OR of 1.54 (95% confidence interval) for risk of psoriasis with PPI exposure.^1^ Nonetheless, in certain subsets of patients, psoriasis may improve with PPI use as in the case of a prospective pilot study demonstrating a mean PASI improvement of 83.9% with esomeprazole monotherapy for a 90 day period.^3^

Study limitations include concomitant medication use and incomplete follow-up. Regardless, we highlight evidence demonstrating that in certain patient subsets, PPI use can induce and/or ameliorate psoriasis. Additional research is warranted regarding this relationship and appropriate monitoring.

## Supplemental Material

sj-docx-1-cms-10.1177_12034754241265711 – Supplemental material for Association of Proton Pump Inhibitors on Psoriasis Treatment and Development: A Systematic Review Supplemental material, sj-docx-1-cms-10.1177_12034754241265711 for Association of Proton Pump Inhibitors on Psoriasis Treatment and Development: A Systematic Review by Siddhartha Sood, Ryan Geng, Samantha Bestavros, Khalad Maliyar, Muskaan Sachdeva, Asfandyar Mufti and Jensen Yeung in Journal of Cutaneous Medicine and Surgery

sj-docx-2-cms-10.1177_12034754241265711 – Supplemental material for Association of Proton Pump Inhibitors on Psoriasis Treatment and Development: A Systematic Review Supplemental material, sj-docx-2-cms-10.1177_12034754241265711 for Association of Proton Pump Inhibitors on Psoriasis Treatment and Development: A Systematic Review by Siddhartha Sood, Ryan Geng, Samantha Bestavros, Khalad Maliyar, Muskaan Sachdeva, Asfandyar Mufti and Jensen Yeung in Journal of Cutaneous Medicine and Surgery

sj-docx-3-cms-10.1177_12034754241265711 – Supplemental material for Association of Proton Pump Inhibitors on Psoriasis Treatment and Development: A Systematic Review Supplemental material, sj-docx-3-cms-10.1177_12034754241265711 for Association of Proton Pump Inhibitors on Psoriasis Treatment and Development: A Systematic Review by Siddhartha Sood, Ryan Geng, Samantha Bestavros, Khalad Maliyar, Muskaan Sachdeva, Asfandyar Mufti and Jensen Yeung in Journal of Cutaneous Medicine and Surgery

sj-docx-4-cms-10.1177_12034754241265711 – Supplemental material for Association of Proton Pump Inhibitors on Psoriasis Treatment and Development: A Systematic Review Supplemental material, sj-docx-4-cms-10.1177_12034754241265711 for Association of Proton Pump Inhibitors on Psoriasis Treatment and Development: A Systematic Review by Siddhartha Sood, Ryan Geng, Samantha Bestavros, Khalad Maliyar, Muskaan Sachdeva, Asfandyar Mufti and Jensen Yeung in Journal of Cutaneous Medicine and Surgery
