# Supplementary material for: Association of Proton Pump Inhibitors on Psoriasis Treatment and Development: A Systematic Review
Source: J Cutan Med Surg. 2024 Jul 26;28(5):502–3. doi: 10.1177/12034754241265711 (PMC11528871; doi:10.1177/12034754241265711)
Supplement: sj-docx-1-cms-10.1177_12034754241265711 – Supplemental material for Association of Proton Pump Inhibitors on Psoriasis Treatment and Development: A Systematic Review [file sj-docx-1-cms-10.1177_12034754241265711.docx]

**
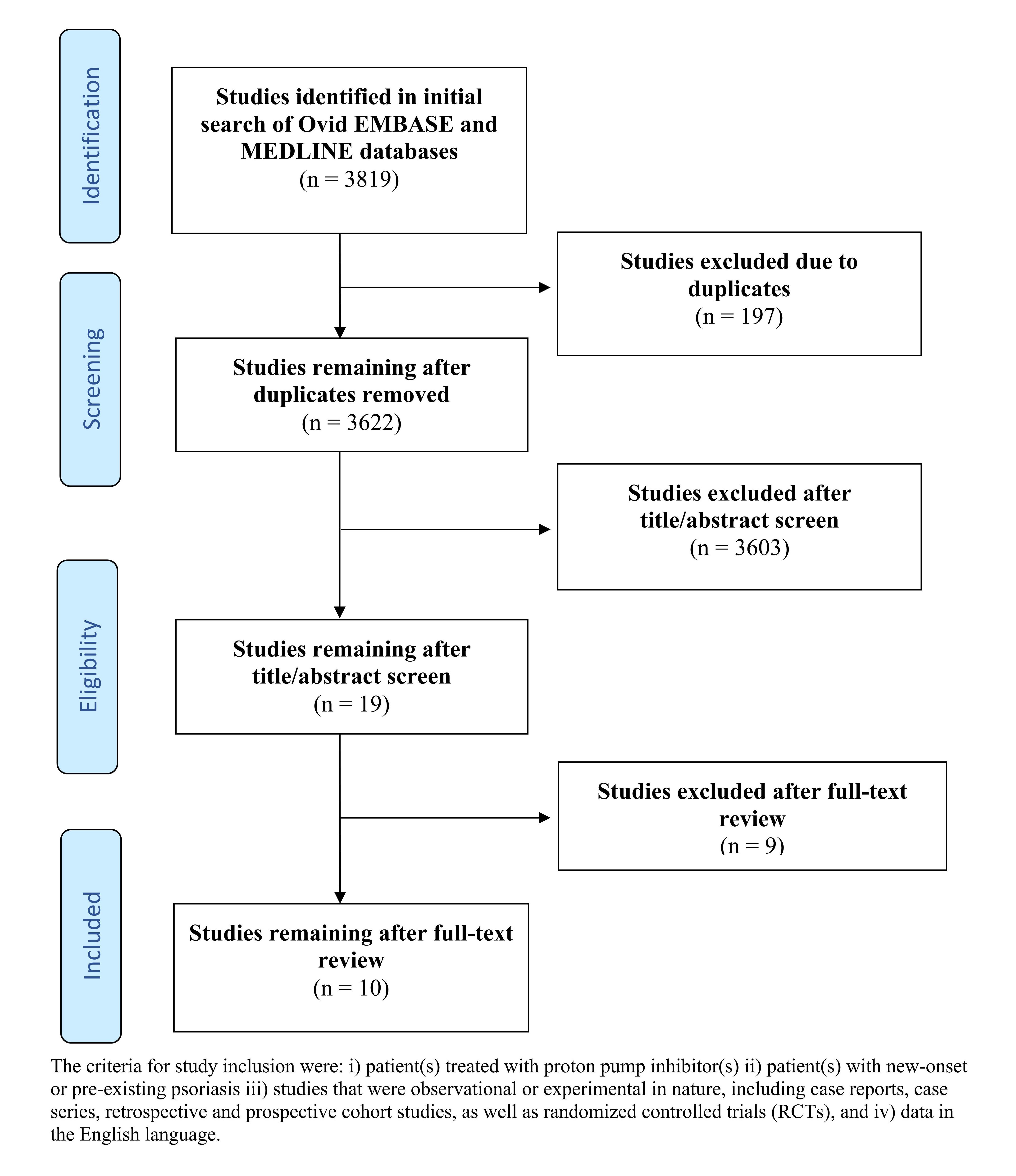
**

**Supplemental Figure 1.** Flow diagram of literature screening using the Preferred Reporting Items for Systematic Reviews and Meta-Analyses (PRISMA) guidelines. Figure adapted from http://prisma-statement.org.
