# Supplementary material for: Association of Proton Pump Inhibitors on Psoriasis Treatment and Development: A Systematic Review
Source: J Cutan Med Surg. 2024 Jul 26;28(5):502–3. doi: 10.1177/12034754241265711 (PMC11528871; doi:10.1177/12034754241265711)
Supplement: sj-docx-2-cms-10.1177_12034754241265711 – Supplemental material for Association of Proton Pump Inhibitors on Psoriasis Treatment and Development: A Systematic Review [file sj-docx-2-cms-10.1177_12034754241265711.docx]

**Supplemental Table 1.** Search strategy used for literature screening.

Database(s): Embase Classic+Embase 1947 to 2024 February 27, Ovid MEDLINE(R) ALL 1946 to 2024 February 27

Search Strategy:

| **#** | **Searches** | **Results** |
| --- | --- | --- |
| 1 | proton pump inhibitor.mp. [mp=ti, ab, hw, tn, ot, dm, mf, dv, kf, fx, dq, bt, nm, ox, px, rx, ui, sy, ux, mx] | 64161 |
| 2 | lansoprazole.mp. [mp=ti, ab, hw, tn, ot, dm, mf, dv, kf, fx, dq, bt, nm, ox, px, rx, ui, sy, ux, mx] | 16638 |
| 3 | omeprazole.mp. [mp=ti, ab, hw, tn, ot, dm, mf, dv, kf, fx, dq, bt, nm, ox, px, rx, ui, sy, ux, mx] | 52383 |
| 4 | pantoprazole.mp. [mp=ti, ab, hw, tn, ot, dm, mf, dv, kf, fx, dq, bt, nm, ox, px, rx, ui, sy, ux, mx] | 17416 |
| 5 | rabeprazole.mp. [mp=ti, ab, hw, tn, ot, dm, mf, dv, kf, fx, dq, bt, nm, ox, px, rx, ui, sy, ux, mx] | 8242 |
| 6 | esomeprazole.mp. [mp=ti, ab, hw, tn, ot, dm, mf, dv, kf, fx, dq, bt, nm, ox, px, rx, ui, sy, ux, mx] | 13049 |
| 7 | autoimmune.mp. [mp=ti, ab, hw, tn, ot, dm, mf, dv, kf, fx, dq, bt, nm, ox, px, rx, ui, sy, ux, mx] | 584650 |
| 8 | cutaneous.mp. [mp=ti, ab, hw, tn, ot, dm, mf, dv, kf, fx, dq, bt, nm, ox, px, rx, ui, sy, ux, mx] | 500283 |
| 9 | psoriasis.mp. [mp=ti, ab, hw, tn, ot, dm, mf, dv, kf, fx, dq, bt, nm, ox, px, rx, ui, sy, ux, mx] | 171812 |
| 10 | psoriasiform.mp. [mp=ti, ab, hw, tn, ot, dm, mf, dv, kf, fx, dq, bt, nm, ox, px, rx, ui, sy, ux, mx] | 4277 |
| 11 | eruption.mp. [mp=ti, ab, hw, tn, ot, dm, mf, dv, kf, fx, dq, bt, nm, ox, px, rx, ui, sy, ux, mx] | 93001 |
| 12 | 1 or 2 or 3 or 4 or 5 or 6 | 125879 |
| 13 | 7 or 8 or 9 or 10 or 11 | 1293202 |
| 14 | 12 and 13 | 4161 |
| 15 | limit 14 to english language | 3997 |
| 16 | limit 15 to humans | 3819 |
| 17 | remove duplicates from 16 | 3622 |
|  |  |  |
