# Supplementary material for: Association of Proton Pump Inhibitors on Psoriasis Treatment and Development: A Systematic Review
Source: J Cutan Med Surg. 2024 Jul 26;28(5):502–3. doi: 10.1177/12034754241265711 (PMC11528871; doi:10.1177/12034754241265711)
Supplement: sj-docx-3-cms-10.1177_12034754241265711 – Supplemental material for Association of Proton Pump Inhibitors on Psoriasis Treatment and Development: A Systematic Review [file sj-docx-3-cms-10.1177_12034754241265711.docx]

**Supplemental Table 2.** Cases of psoriasis induced by or treated using proton pump inhibitor(s).

Abbreviations: BID, twice a day; CC, complete clearance; CoH, prospective cohort study; CRS, case report; F, female; GI, gastrointestinal; HR, hazard ratio; IM, improvement; M, male; N, no; N/A, not applicable; NB-UVB, narrowband ultraviolet B phototherapy; NIM, no improvement; NR, not reported; OR, odds ratio; PASI, Psoriasis Area and Severity Index; PASI 50, 50% improvement from baseline in PASI; PASI 75, 75% improvement from baseline in PASI; PASI 90, 90% improvement from baseline in PASI; PO, oral; PR, partial resolution; QD, once daily; RS, retrospective study; SC, subcutaneous; TOP, topical; Y, yes

| **Study** | **Study design (level of evidence)** | **Sample size** | **Mean age (range)** | **Sex** | **Comorbidities (n)** | **Mean duration of psoriasis, months** | **Type of psoriasis** | **Concurrent psoriasis medications [route, dose, and frequency] (n)** | **Concurrent non-psoriasis therapies [route, dose, and frequency]** | **PPI indication** | **H. pylori infection? (Y/N)** | **Proton pump inhibitor(s) [route, dose, and frequency] (n)** | **Progression of psoriasis [DE NOVO, IM, NIM] (n)** | **Measures of association for psoriasis and PPI (e.g. IR, OR)** | **Outcomes of PPI use [CC, PR, NIM]** | **PASI (pre-treatment/post-)** | **Mean change in PASI, %** | **Number of PASI 50, PASI 75, PASI 90 responders (n)** | **Treatment duration, days** | **Recurrence following initial resolution (Y/N) (n)** | **Follow-up period, months** |
| --- | --- | --- | --- | --- | --- | --- | --- | --- | --- | --- | --- | --- | --- | --- | --- | --- | --- | --- | --- | --- | --- |
| Ahmed 2021^1^ | RS (2b) | 20 | 34.7 | M(16); F(4) | NR | NR | Plaque (20) | Acitretin [PO, 10mg, NR] (NR); calcipotriol [TOP, 50ug, NR] (NR); CS: betamethasone dipropionate [TOP, 0.5mg, NR] (NR); cyclosporine [25-100mg, NR] (NR); etanercept [SC, 25-50mg, NR] (NR); MTX [PO, 2.5mg, NR] (NR); tacrolimus [TOP, 0.1%, NR] (NR); tofacitinib [PO, 5mg, NR] (NR); ustekinumab [SC, 130mg, NR] (NR); NB-UVB (NR) | Amoxicillin [PO, 1g BID]; clarithromycin [PO, 500mg, BID] | Peptic ulcer | Y (20) | Esomeprazole [PO, 40mg, QD] (NR); unknown PPI [PO, higher dose, BID] (NR) | IM (20) | NR | PR | 31.8 / NR | NR | NR | 14 | N | 1.5 |
| Ali 2008^2^ | CRS (5) | 1 | 48 | F | NR | 180 | Plaque | None | Amoxicillin [PO, 1g BID]; clarithromycin [PO, 500mg, BID] | Peptic ulcer | Y | Lansoprazole [PO, 30mg, BID] (1) | IM (1) | NR | CC | NR / NR | NR | NR | 7 | N | 0.5 |
| Bafutto 2019^3^ | CoH (2b) | 10 | 52.1 | M(5);F(5) | NR | NR | Plaque (10) | None | None | Psoriasis | NR | Esomeprazole [PO, 40mg, BID] (10) | IM (10) | NR | PR | 5.52 / 0.89 | -83.9 | PASI 50 (10); PASI 75 (7); PASI 90 (6) | 90 | N | 3 |
| Bardazzi 2021^4^ | CoH (2b) | 42 | 53 | M(25);F(17) | NR | 360 | Plaque (42) | Adalimumab [SC, NR, NR] (18); etanercept [SC, NR, NR] (8); golimumab [SC, NR, NR] (2); ustekinumab [SC, NR, NR] (13) | Amoxicillin [PO, 1g, BID]; clarithromycin [PO, 500mg, BID]; tinidazole [PO, 500mg, BID] | Peptic ulcer | Y (42) | Esomeprazole [PO, 40mg, BID] (42) | IM (42) | NR | PR | 4.02 / 2.36 | -41.3 | NR | 10 | N | 6 |
| Campanati 2015^5^ | CoH (2b) | 43 | 49 | M(25);F(18) | NR | NR | Plaque (32); guttate (4); pustular (3); palmoplantar (1) | PUVA (43) | Amoxicillin [PO, 1g BID]; clarithromycin [PO, 500mg, BID] | Peptic ulcer | Y (43) | Esomeprazole [PO, 40mg, BID] (43) | IM (43) | NR | PR | 17.9 / 8.36 | -53.3 | NR | 7 | N | 1 |
| Dauden 2000^6^ | CoH (2b) | 10 | NR | NR | NR | NR | NR | NR | Amoxicillin [PO, 1g BID]; clarithromycin [PO, 500mg, BID] | Peptic ulcer | Y (10) | Omeprazole [PO, NR, NR] (62) | NIM (10) | NR | NIM | NR / NR | NR | NR | 7 | N | 12 |
| Hubner 2008^7^ | CRS (5) | 1 | 35 | M | Psoriatic arthritis | 72 | Palmoplantar (1) | Betamethasone dipropionate [TOP, 0.05%, QD]; calcipotriol [TOP, 50ug/g, QD] | Amoxicillin [PO, 1g BID]; clarithromycin [PO, 500mg, BID] | Peptic ulcer | Y | Omeprazole [PO, 20mg, BID] (1) | IM (1) | NR | CC | NR / 0 | 100 | PASI 50 (1); PASI 75 (1); PASI 90 (1) | 7 | N | 36 |
| Li 2021^8^ | RS (2b) | 2878 | 50.44 | M(1715);F(1163) | Infectious disease (1052); hypertension (704); diabetes (362); dyslipidemia (348); anxious disorder (205); alcohol use disorder (100); autoimmune disease (90); depressive disorder (90); obesity (10) | NR | NR | NR | NR | Peptic ulcer (2833), GERD (1313); upper GI bleed (1256) | NR | Lansoprazole [PO, NR, NR] (824); omeprazole [PO, NR, NR] (682); esomeprazole [PO, NR, NR] (575); pantoprazole [PO, NR, NR] (347); rabeprazole [PO, NR, NR] (218); unknown (232) | DE NOVO (2878) | Overall (OR: 1.54 to 1.56); lansoprazole (OR: 1.25); rabeprazole (OR: 1.18); esomeprazole (OR: 1.11); omeprazole (OR: 1.08); pantoprazole (OR: 1.07) | N/A | N/A | N/A | NR | N/A | N/A | N/A |
| Lin 2021^9^ | RS (2b) | 12 | NR | NR | NR | NR | NR | NR | NR | NR | NR | Lansoprazole [PO, NR, NR] (NR); omeprazole [PO, NR, NR] (NR); esomeprazole [PO, NR, NR] (NR); pantoprazole [PO, NR, NR] (NR); rabeprazole [PO, NR, NR] (NR) | DE NOVO (12) | Overall (adjusted HR: 2.568) | N/A | N/A | N/A | NR | N/A | N/A | N/A |
| Onsun 2012^10^ | CoH (2b) | 50 of 75 | NR | NR | NR | NR | Plaque (50) | Acitretin [PO, 25mg, QD] (50) | Amoxicillin [PO, 1g BID]; clarithromycin [PO, 500mg, BID] | Peptic ulcer | Y (50) | Lansoprazole [PO, 30mg, BID] (50) | IM (50) | NR | PR | 4.32 / 0.94 | -78.2 | NR | 14 | N | 2 |
| Onsun 2012^10^ | CoH (2b) | 25 of 75 | NR | NR | NR | NR | Plaque (25) | None | Amoxicillin [PO, 1g BID]; clarithromycin [PO, 500mg, BID] | Peptic ulcer | Y (25) | Lansoprazole [PO, 30mg, BID] (25) | IM (25) | NR | PR | 2.56 / 1.34 | -47.7 | NR | 14 | N | 2 |

References:

1. Ahmed AS, Al-Najjar AH, Alshalahi H, Altowayan WM, Elgharabawy RM. Clinical Significance of *Helicobacter pylori* Infection on Psoriasis Severity. *J Interferon Cytokine Res*. 2021;41(2):44-51. doi:10.1089/jir.2020.0144
2. Ali M, Whitehead M. Clearance of chronic psoriasis after eradication therapy for Helicobacter pylori infection. J Eur Acad Dermatol Venereol. 2008;22(6):753-754. doi:10.1111/j.1468-3083.2007.02452.x
3. Bafutto M, Oliveira EC, Zaterka S. EVALUATION OF PSORIASIS TREATMENT WITH ESOMEPRAZOLE - A PILOT STUDY. Arq Gastroenterol. 2019;56(3):261-263. Published 2019 Sep 30. doi:10.1590/S0004-2803.201900000-49
4. Bardazzi F, Magnano M, Fiorini G, et al. Helicobacter pylori infection in psoriatic patients during biological therapy. *Ital J Dermatol Venerol*. 2021;156(5):570-574. doi:10.23736/S2784-8671.19.06410-1
5. Campanati A, Ganzetti G, Martina E, et al. Helicobacter pylori infection in psoriasis: results of a clinical study and review of the literature. *Int J Dermatol*. 2015;54(5):e109-e114. doi:10.1111/ijd.12798
6. Daudén E, Vázquez-Carrasco MA, Peñas PF, Pajares JM, García-Díez A. Association of Helicobacter pylori infection with psoriasis and lichen planus: prevalence and effect of eradication therapy. *Arch Dermatol*. 2000;136(10):1275-1276. doi:10.1001/archderm.136.10.1275
7. Martin Hübner A, Tenbaum SP. Complete remission of palmoplantar psoriasis through Helicobacter pylori eradication: a case report. *Clin Exp Dermatol*. 2008;33(3):339-340. doi:10.1111/j.1365-2230.2007.02634.x
8. Li CY, Dai YX, Chang YT, et al. Proton Pump Inhibitors Are Associated with Increased Risk of Psoriasis: A Nationwide Nested Case-Control Study. *Dermatology*. 2021;237(6):884-890. doi:10.1159/000517515
9. Lin SH, Chang YS, Lin TM, et al. Proton Pump Inhibitors Increase the Risk of Autoimmune Diseases: A Nationwide Cohort Study. *Front Immunol*. 2021;12:736036. Published 2021 Sep 30. doi:10.3389/fimmu.2021.736036
10. Onsun N, Arda Ulusal H, Su O, Beycan I, Biyik Ozkaya D, Senocak M. Impact of Helicobacter pylori infection on severity of psoriasis and response to treatment. *Eur J Dermatol*. 2012;22(1):117-120. doi:10.1684/ejd.2011.1579
