# Supplementary material for: Association of Proton Pump Inhibitors on Psoriasis Treatment and Development: A Systematic Review
Source: J Cutan Med Surg. 2024 Jul 26;28(5):502–3. doi: 10.1177/12034754241265711 (PMC11528871; doi:10.1177/12034754241265711)
Supplement: sj-docx-4-cms-10.1177_12034754241265711 – Supplemental material for Association of Proton Pump Inhibitors on Psoriasis Treatment and Development: A Systematic Review [file sj-docx-4-cms-10.1177_12034754241265711.docx]

**Supplemental Table 3.** Summary of proton pump inhibitor use and psoriasis.

| **Study design (n/N, %)** |  |
| --- | --- |
| Prospective cohort studies | 5/10 (50%) |
| Retrospective studies | 3/10 (30%) |
| Case reports | 2/10 (20%) |
| **Outcomes of PPI use (n/N, %)** |  |
| *De novo* psoriasis | 2890/3092 (93.5%) |
| IM of psoriasis | 192/3092 (6%) |
| NIM of psoriasis | 10/3092 (2.6%) |
| **Types of PPIs (n/N, %)** |  |
| Lansoprazole | 900/2533 (35.5%) |
| Omeprazole | 745/2533 (29.4%) |
| Esomeprazole | 670/2533 (26.5%) |
| **Change in PASI from baseline (%)** | |
| Mean (reported cases) | -58.9% (171/3092) |
| Range | -41.3% to -100% |
| PASI 50 responders (n/N, %) | 11/11 (100%) |
| PASI 75 responders (n/N, %) | 8/11 (72.7%) |
| PASI 90 responders (n/N, %) | 7/11 (63.6%) |
| **Treatment outcomes** | |
| CC (n/N, %) | 2/202 (1%) |
| *Mean treatment duration for CC (reported cases) (days)* | 7 (2/2) |
| PR (n/N, %) | 190/202 (94%) |
| *Mean treatment duration for PR (reported cases) (days)* | 15.5 (190/190) |
| NIM (n/N, %) | 10/202 (5%) |
| *Mean treatment duration for NIM (reported cases) (days)* | 7 (10/10) |
| **Treatment characteristics** | |
| Treatment duration (days): Mean | 15 |
| Treatment duration (days): Range | 7 to 90 |
| Follow-up period (months): Mean | 3.3 |
| Follow-up period (months): Range | 0.5 to 12 |
| Abbreviations: CC, complete clearance; IM, improvement; NIM, no improvement; PASI, Psoriasis Area and Severity Index; PPI, proton pump inhibitor; PR, partial resolution. | |
|  | |
